# New insights into Lyme disease

**DOI:** 10.1016/j.redox.2015.03.002

**Published:** 2015-03-16

**Authors:** Brandon N. Peacock, Teshome B. Gherezghiher, Jennifer D. Hilario, Gottfried H. Kellermann

**Affiliations:** Pharmasan Labs, Inc., Osceola, WI, USA

**Keywords:** Lyme, Oxidative stress, Inflammation, Cytosolic calcium, Mitochondria, PBMCs, peripheral blood mononuclear cells, HBSS, Hanks' balanced salt solution, ROS, reactive oxygen species, RNS, reactive nitrogen species, SOD, superoxide dismutase, CDC, centers for disease control and prevention, ELISA, enzyme-linked immunosorbent assay, EM, erythema migrans, fMLP, formyl-methionyl-leucyl-ribose, IL, interleukin, TNFα, tumor necrosis factor alpha, IFNγ, interferon gamma, TGF β, transforming growth factor beta, NF-κB, nuclear factor Kappa-light-chain-enhancer of activated B cells

## Abstract

Lyme borreliosis is transmitted through the bite of a tick that is infected by the bacterial spirochete *Borrelia burgdorferi*. Clinical manifestation of the disease can lead to heart conditions, neurological disorders, and inflammatory disorders. Oxidative stress has been implicated in the pathogenesis of many human diseases. The aim of this study was to investigate the mechanisms of oxidative stress and intracellular communication in Lyme borreliosis patients. Mitochondrial superoxide and cytosolic ionized calcium was measured in peripheral blood mononuclear cells (PBMCs) of Lyme borreliosis patients and healthy controls. Mitochondrial superoxide levels were significantly higher (*p*<0.0001) in Lyme borreliosis patients (*n*=32) as compared to healthy controls (*n*=30). Significantly low (*p*<0.0001) levels of cytosolic ionized calcium were also observed in Lyme borreliosis patients (*n*=11) when compared to healthy controls (*n*=11). These results indicate that there is an imbalance of reactive oxygen species and cytosolic calcium in Lyme borreliosis patients. The results further suggest that oxidative stress and interrupted intracellular communication may ultimately contribute to a condition of mitochondrial dysfunction in the immune cells of Lyme borreliosis patients.

## Highlights

• Positivity of *Borrelia burgdorferi* infection was assessed by a Lyme ELISPOT assay.
• Assessed levels of mitochondrial superoxide and cytosolic calcium in patient PBMCs.
• Lyme borreliosis patients showed a marked increase in mitochondrial superoxide.
• Levels of cytosolic calcium were significantly lower in Lyme borreliosis patients.
• Suggesting that Lyme borreliosis may lead to a state of mitochondrial dysfunction.

## Graphical abstract

**Figure:**
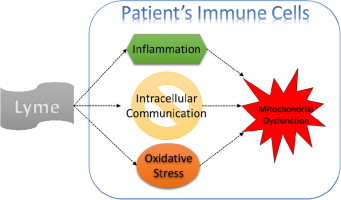

## Introduction

In North America, *Borrelia burgdorferi* is the predominant bacterial species responsible for infection leading to the emerging health threat of Lyme borreliosis (Lyme disease) [1,2]. In 2013 there were more than twenty-five thousand new cases of Lyme borreliosis reported by the Centers for Disease Control and Prevention (CDC) across the United States [3]. The currently accepted practice for clinical diagnosis of Lyme disease is using the two-tier testing of ELISA and Western Blot analysis [4]. These tests are limited in both sensitivity and specificity, often providing both false negative and false positive results [5–8]. To overcome this limitation, our laboratory has developed an enhanced T cell-based immunospot assay which bridges the gap between the ability to detect humoral immunity and cellular immunity to *B. burgdorferi*. We have been able to simultaneously increase the number of true positive diagnoses while decreasing the number of false positive and negative results [9].

When an infected tick bites a host, *B. burgdorferi* is transmitted through the infected tick's saliva. Once transferred, *B. burgdorferi* stimulates the host's immune system to activate a localized inflammatory response [10]. Consequently the infection often presents itself by the presence of a “bulls-eye” rash called erythema migrans (EM) within 3–30 days post infection [11]. Once infected, *B. burgdorferi* disseminates and causes a variety of immunological and inflammatory reactions throughout the body. Early manifestations of infection can lead to heart complications (e.g. carditis, dizziness, palpitations), neurological disorders (e.g. Bell's and/or cranial nerve palsy, peripheral neuropathy), and other inflammatory disorders (e.g. head and neck aches (meningitis), arthritis) [11]. If treatment is ineffective (Post-Treatment Lyme Disease Syndrome) or if infected individuals remain undiagnosed and untreated, some symptoms can persist for months to years. These symptoms may include muscular pains, arthritis, neurological disorders, fatigue, etc. [10,11].

In order to combat an infection the host's immune cells will generate reactive oxygen species (ROS) through NADPH Oxidase (NOX; producing superoxide anion radical), and nitric oxide synthase (NOS; producing nitric oxide) [12]. The predominant generator of ROS within the cells is the mitochondria and it is believed that the major contributor to cellular oxidative damage is mitochondrial superoxide [13]. Upon the generation of superoxide (O_2_^−•^) and nitric oxide (NO), the reactive nitrogen species (RNS) peroxynitrite (ONOO^−^) can be formed. An example of this process is the neutrophil defense mechanism of oxidative burst which results in the mobilization of calcium and activation of NADPH oxidase leading to the subsequent generation of superoxide. Superoxide dismutase (SOD) then converts superoxide to hydrogen peroxide (H_2_O_2_), which is bactericidal [14]. These reactive species are normally kept in balance by endogenous antioxidant enzymes such as SOD and glutathione peroxidase which converts H_2_O_2_ to water [15]. However, if an imbalance occurs between ROS/RNS and the antioxidant enzymes, oxidative stress will ensue causing a toxic environment that can lead to damage of DNA, protein, and lipids [16]. In addition to creating a toxic environment for pathogens, ROS and RNS activate NF-κB. One of the major roles of the NF-κB pathway is generation of pro-inflammatory cytokines such as Interleukins 1 & 6, TNFα, and IFNγ [17]. Individuals infected by *B. burgdorferi* present with significantly increased levels of TNFα in their sera and synovial fluid [18,19]. This observation is similar to what is found in patients diagnosed with rheumatoid, suppurative, and reactive arthritis [19]. A number of studies have shown that in vitro stimulation of an infected individuals immune cells by either *B. burgdorferi* or associated proteins results in an induction of pro-inflammatory cytokines (IL-1β, IL-6, IL-17, IL-23, TNFα, and TGF β) [19–22]. Importantly, studies have shown that during an active Lyme infection or *with* in vitro stimulation with *B. burgdorferi*, both NOS and ROS are generated [22,23].

However, the specific mechanisms of how these reactive species interact with and change intracellular communication of immune cells during an infection by *B. burgdorferi* are still unknown. In a previous study we addressed antigen specific T cell response to *B. burgdorferi* by measuring release of IFNγ [9]. The goal of this study was to explore the immune stimulated, inflammatory response to the oxidative stress state in PBMCs of Lyme borreliosis patients. To accomplish this we compared levels of mitochondrial superoxide and cytosolic ionized calcium in Lyme borreliosis patients with those in healthy controls.

## Materials and methods

### Reagents

Unless otherwise stated, all reagents were purchased from Sigma Aldrich (St. Louis, MO).

### Clinical study population

Healthy control subjects in this study were either healthy adults without known inflammatory conditions or history of *Borrelia* infection. Subjects suspected for Lyme borreliosis infection were classified by CDC surveillance definition of Lyme disease, including clinical signs and symptoms, history of possible exposure to infected blacklegged ticks, with or without a positive antibody response to B. burgdorferi by ELISA and Western Blot, interpreted according to CDC and the Infectious Disease Society of America (IDSA) criteria [11]. In addition, any subject known to be on antibiotic therapy was omitted from this study. Study subjects were tested further to confirm their negativity or positivity of *B. burgdorferi* infection by a Lyme ELISpot assay [9]. Collection of blood, isolation of PBMCs, and determination of infection by *B. burgdorferi* were all performed as described previously [9]. All individuals gave their informed consent. The studies were performed following a protocol approved by the internal clinical ethics committee.

### Mitochondrial superoxide

Levels of mitochondrial superoxide, in PBMCs, were measured using the fluorogenic dye MitoSOXTM Red (Life technologies; Eugen, OR). Measurements were made following the manufacturer's suggested protocol with slight modifications. Isolated PBMCs were incubated with MitoSOXTM Red for 10 min at 37 °C with 5% CO_2_ at a ratio of 5 fmol MitoSOX per Cell, in Hanks’ Balanced Salt Solution (HBSS). Cells were then washed once in HBSS at room temperature with centrifugation at 250*g* for 5 min followed by resuspension in HBSS. Fluorescence was measured at 37 °C with an excitation *λ* of 510 nm and an emission *λ* at 595 nm.

### Cytosolic ionized calcium

Cytosolic ionized calcium (Ca^2+^_I_) levels were measured in PBMCs by using the calcium specific fluorogenic dye Fura2 AM ester (Molecular Probes/Life technologies; Eugen, OR). Measurements were made following the manufacturer's suggested protocol and for details read work of Carruthers et al. [27]. Briefly, isolated PBMCs were incubated with Fura2 for 30 min at room temperature at a ratio of 0.5 ƒmol Fura2 per Cell, in HBSS. Cells were then washed three times in HBSS, at room temperature, with centrifugation at 250*g* for 5 min, once the washes were completed cells were resuspended in HBSS and incubated at room temperature for 10 min to allow de-esterification of AM ester by intracellular esterase. Cells were washed three times again as before to remove any dye that may have leaked. Fluorescence was measured at room temperature with an excitation *λ* of either 335 nm (Ca^2+^_I_ Bound Fura2) or 363 nm (Ca^2+^_I_ Free Fura2) and an emission *λ* being read at 510 nm. Calibrators were used for each subject to measure the minimal (Ca^2+^_I_ Free Fura2) and maximal (Ca^2+^_I_ Bound Fura2) signals. The calibration for measuring the minimal level was achieved by post-de-esterification addition of 10 mM EGTA and 0.05% Triton X100. The calibration for measuring the maximal level was achieved by post-de-esterification addition of 5 µM Ionomycin and 20 mM Calcium. Assuming the *K_d_* of Ca^2+^_I_ -Fura2 at room temperature in the cytosol to be 143 nM the equation suggested by the manufacturer was used to determine [Ca^2+^_I_].

### Statistical analysis

Student's *t* test was used to compare results of healthy controls with Lyme borreliosis patients. A *p*-value of <0.05 was considered statistically significant. The analyses were done by Prism 6.0 analysis software (GraphPad Software Inc., La Jolla, CA).

## Results and discussion

**Fig. 1:**
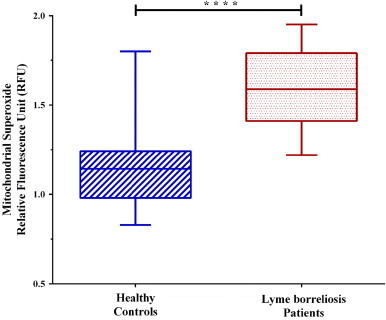
Mitochondrial superoxide levels of PBMCs. Dashed blue lines (left) represent healthy controls (*x̄*=1.14 RFU), where red dots (right) represent Lyme borreliosis patients (*x̄*=1.59 RFU). Significant difference between groups was measured by Student's *t*-test (****p<0.0001). Box-and-whisker plots are represented with max/min outliers, 25th and 75th on the hinges, and middle line representing the median.

Excessive mitochondrial superoxide is believed to be one of the main contributors to oxidative stress and damage within the cell. Oxidative stress can damage DNA, proteins, and lipids, this damage has been proposed to contribute to diseases and disorders such as cancer, Parkinson's, Alzheimer's, atherosclerosis, chronic fatigue syndrome, and possibly Lyme borreliosis [12,17]. Our goal was to assess if mitochondrial superoxide is a contributing factor to oxidative stress within PBMCs of Lyme borreliosis patients (Table 1A). We measured the level of mitochondrial superoxide in PBMCs using the mitochondrial targeted and superoxide specific fluorogenic dye MitoSOXTM Red. Significantly higher levels of mitochondrial superoxide were observed in Lyme borreliosis patients when compared to healthy controls (Fig. 1).

A feed forward-cycle during pathophysiological conditions has been proposed by Dikalov et al. in which they suggest that “NADPH oxidases increase mitochondrial ROS, which further activates cytoplasmic NADPH oxidases and increases cellular superoxide production…” [24]. This cycle could be the link between our observation of significantly increased mitochondrial superoxide and *B. burgdorferi* induced activation of NADPH oxidase resulting in oxidative burst [23]. Dikalov also cites findings that by reducing mitochondrial ROS a resulting down regulation of NADPH oxidase occurred which ultimately broke the cycle causing oxidative stress in the cell [24]. As mentioned in the introduction, the cell has enzymatic antioxidants that serve this function. However, *B. burgdorferi* has been shown to passively absorb the host's cysteine [25]. Cysteine is one of the main amino acids required to synthesize glutathione (GSH), so depletion of cysteine concomitantly lowers the levels of glutathione in the host, in which GSH is a powerful antioxidant that plays a critical role in scavenging excess ROS and RNS (Fig. 3).

**Fig. 2:**
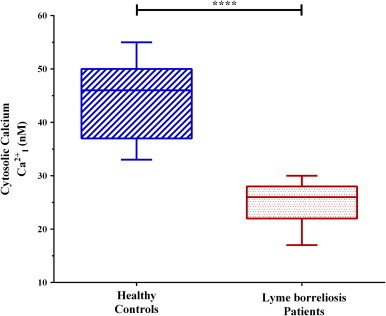
Cytosolic ionized calcium levels of PBMCs. Dashed blue lines (left) represent healthy controls (*x̄*=46 nM), where red dots (right) represent Lyme borreliosis patients (*x̄*=26 nM). Significant difference between groups was measured by Student's *t*-test (****p<0.0001). Box-and-whisker plots are represented with max/min outliers, 25th and 75th on the hinges, and middle line representing the median.

Another integral molecule of cellular communication is ionized calcium (Ca^2+^_I_). Ionized calcium has a plethora of roles within the cell including maintenance of the cellular and mitochondrial membrane potentials, gene regulation, cell proliferation, and apoptosis. The level of Ca^2+^ in the cytosol is highly regulated and to maintain homeostasis reserves are stored in the endoplasmic reticulum or mitochondria [26]. This homeostasis can fluctuate in response to inflammation or infection. In regards to inflammation, rheumatoid arthritis patient’s PBMCs present with an approximate 22% decrease in resting cytosolic calcium when compared to un-afflicted patients [27]. However, an infection can stimulate neutrophils to activate NADPH oxidase which requires the mobilization of calcium. Since Lyme borreliosis is an infection that can lead to a severe inflammatory state, we assessed the levels of cytosolic Ca^2+^_I_ in infected patient PBMCs compared with uninfected individuals (Table 1B). Our observations (Fig. 2) have shown a significant decrease in the levels of cytosolic Ca^2+^_I_ in PBMCs of Lyme borreliosis patients when compared to healthy controls.

**Fig. 3:**
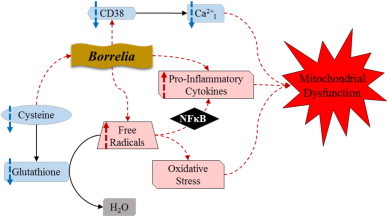
Proposed scheme of the effect of *Borrelia* infection on metabolic and signaling pathways within host cells. This scheme shows the cells normal processes (solid arrows) used to scavenge free radicals and maintain calcium homeostasis. The proposed effect of *Borrelia* infection is shown (dashed arrows) by the induced state of oxidative stress, disrupted calcium homeostasis, increased pro-inflammatory cytokines, and ultimately, mitochondrial dysfunction.

Besides the role of Ca^2+^_I_ mobilization in NADPH activation, it is also necessary for chemotaxis and cell migration [28]. Chemotaxis is mediated by cyclic ADP-ribose (cADPR) which regulates intracellular calcium release. Sanchez et al. have shown that the chemoattractant formyl-methionyl-leucyl-ribose (fMLP) released by bacteria initiates chemotaxis of neutrophils by stimulating cADPR to induce intracellular Ca^2+^ release and sustains extracellular calcium influx [28]. The synthesis and hydrolysis of cADPR from nicotinamide adenine dinucleotide (NAD^+^) is catalyzed by cyclic ADP ribose hydrolase (CD38) [29]. A recent study has shown the effect of Lyme borreliosis on chemotaxis and migration of dendritic cells (DC) through interference of DC CD38 expression, resulting in an almost negligible level of CD38 protein [30]. These findings suggest that *Borrelia* may inhibit dendritic cell migration to lymph nodes through limiting calcium mobility and ultimately inhibiting further response by the host's immune cells to the site of infection [30]. This could possibly explain the significant decrease in levels of cytosolic Ca^2+^_I_ we observed in Lyme borreliosis patient PBMCs (Fig. 3).

Another possible effect of *B. burgdorferi* infection may be an alteration to the mitochondrial density of infected cells. Studies of other bacterial infections have shown that there is a similar outcome of oxidative stress and mitochondrial dysfunction in infected cells, but the mitochondrial density remains unchanged. Suliman et al. have shown that when rats were injected with *Escherichia coli* Lipopolysaccharides (LPS), their cardiac cells showed signs of LPS-induce oxidative stress, but there was no change in the mitochondrial density [31]. Garrabou et al. have shown similar findings with PBMCs of septic patients. They found significant evidence of mitochondrial dysfunction and oxidative stress in these patient's PBMCs, but the number of mitochondria remained unaltered [32]. The effect of infection by *B. burgdorferi* on mitochondrial density is unknown, but considering the aforementioned studies, we speculate that a change in the mitochondrial density is unlikely.

In conclusion, our results have shown a significant rise in mitochondrial superoxide, indicative of a state of oxidative stress in the PBMCs of Lyme borreliosis patients. In these same patients we have presented evidence of a significant decrease in levels of cytosolic ionized calcium in PBMCs. Taken together, we hypothesize that these imbalances could cause oxidative stress, depolarization of the mitochondrial membrane, disruption of intracellular communication, and a release of pro-inflammatory cytokines [33]. All of which could ultimately contribute to a condition of mitochondrial dysfunction (Fig. 3). It is our intent to explore this mechanism in Lyme borreliosis patients further by expanding on our preliminary data and assessing additional markers for oxidative stress, intracellular communication, and the inflammatory pathways.

## Conflicts of interest

B.N.P, T.B.G., J.D.H. and G.H.K are employed by Pharmasan Labs, Inc. that develops and offers diagnostic tests.

## Figures and Tables

**Table 1:** Characteristics of the cohorts used in each analysis.

Analysis	Demographic		Healthy controls	Lyme borreliosis patients
A. Mitochondrial superoxide	Sex	Male	6	9
		Female	24	23
	Age	Median ± SD	40±15 (10–75)	40±19 (8–79)
B. Cytosolic calcium	Sex	Male	3	3
		Female	8	8
	Age	Median ± SD	40±12 (25–58)	41±15 (23–69)

## References

[1] Burgdorfer W., Barbour A.G., Hayes S.F., Benach J.L., Grunwaldt E., Davis J.P. (1982). Lyme disease-a tick-borne spirochetosis?. Science.

[2] Steere A.C., Coburn J., Glickstein L. (2004). The emergence of Lyme disease. Journal of Clinical Investigation.

[3] Centers for Disease Control and Prevention, Reported cases of Lyme disease by year, United States, 1995–2013. Available online: http://www.cdc.gov/lyme/stats/chartstables/casesbyyear.html (accessed 29.01.15).

[4] Aguero-Rosenfeld M.E., Wang G., Schwartz I., Wormser G.P. (2005). Diagnosis of lyme borreliosis. Clinical Microbiology Reviews.

[5] Seriburi V., Ndukwe N., Chang Z., Cox M.E., Wormser G.P. (2012). High frequency of false positive IgM immunoblots for Borrelia burgdorferi in clinical practice. Clinical Microbiology and Infection.

[6] Brown S.L., Hansen S.L., Langone J.J. (1999). Role of serology in the diagnosis of Lyme disease. Journal of the American Medical Association.

[7] Brunner M. (2001). New method for detection of Borrelia burgdorferi antigen complexed to antibody in seronegative Lyme disease. Journal of Immunology Methods.

[8] Tylewska-Wierzbanowska S., Chmielewski T. (2002). Limitation of serological testing for Lyme borreliosis: evaluation of ELISA and western blot in comparison with PCR and culture methods. Wiener Klinische Wochenschrift.

[9] Jin C., Roen D.R., Lehmann P.V., Kellermann G.H. (2013). An enhanced ELISPOT assay for sensitive detection of antigen-specific T cell responses to Borrelia burgdorferi. Cells.

[10] Auwaerter P.G., Aucott J., Dumler J.S. (2004). Lyme borreliosis (Lyme disease): molecular and cellular pathobiology and prospects for prevention, diagnosis and treatment. Expert Reviews in Molecular Medicine.

[11] Centers for Disease Control and Prevention, Signs and symptoms of Lyme disease. Available online: http://www.cdc.gov/lyme/signs_symptoms/index.html (accessed 07.08.14).

[12] Pohanka M. (2013). Role of oxidative stress in infectious diseases. A review. Folia Microbiologica.

[13] Brand M.D., Affourtit C., Esteves T.C., Green K., Lambert A.J., Miwa S. (2004). Mitochondrial superoxide: production, biological effects, and activation of uncoupling proteins. Free Radical Biology and Medicine.

[14] Yamashita K., Arai T., Fukuda K., Mori H., Ishii H., Nishioka M. (2001). 6-Formylpterin intracellularly generates hydrogen peroxide and restores the impaired bactericidal activity of human neutrophils. Biochemical and Biophysical Research Communications.

[15] Schafer F.Q., Buettner G.R. (2001). Redox environment of the cell as viewed through the redox state of the glutathione disulfide/glutathione couple. Free Radical Biology and Medicine.

[16] Percário S., Moreira D.R., Gomes B.A., Ferreira M.E., Gonçalves A.C., Laurindo P.S., Vilhena T.C., Dolabela M.F., Green M.D. (2012). Oxidative stress in malaria. International Journal of Molecular Sciences.

[17] Pall M.L. (2001). Common etiology of posttraumatic stress disorder, fibromyalgia, chronic fatigue syndrome and multiple chemical sensitivity via elevated nitric oxide/peroxynitrite. Medical Hypotheses.

[18] Hopkins S.J., Meager A. (1988). Cytokines in synovial fluid: II. The presence of tumour necrosis factor and interferon. Clinical and Experimental Immunology.

[19] Defosse D.L., Johnson R.C. (1992). In vitro and in vivo induction of tumor necrosis factor alpha by Borrelia burgdorferi. Infection and Immunity.

[20] Miller L.C., Isa S., Vannier E., Georgilis K., Steere A.C., Dinarello C.A. (1992). Live Borrelia burgdorferi preferentially activate interleukin-1beta gene expression and protein synthesis over the interleukin-1 receptor antagonist. Journal of Clinical Investigation.

[21] Codolo G., Amedei A., Steere A.C., Papinutto E., Cappon A., Polenghi A. (2008). Borrelia burgdorferi NapA-driven Th17 cell inflammation in lyme arthritis. Arthritis and Rheumatology.

[22] Seiler K.P., Vavrin Z., Eichwald E., Hibbs J.B., Weis J.J. (1995). Nitric oxide production during murine Lyme disease: lack of involvement in host resistance or pathology. Infection and Immunity.

[23] Suhonen J., Hartiala K., Tuominen-Gustafsson H., Viljanen M.K. (2000). Borrelia burgdorferi--induced oxidative burst, calcium mobilization, and phagocytosis of human neutrophils are complement dependent. Journal of Infectious Diseases.

[24] Dikalov S. (2011). Cross talk between mitochondria and NADPH oxidases. Free Radical Biology and Medicine.

[25] Sambri V., Cevenini R. (1992). Incorporation of cysteine by Borrelia burgdorferi and Borrelia hermsii. Canadian Journal of Microbiology.

[26] Clapham D.E. (2007). Calcium signaling. Cell.

[27] Carruthers D.M., Naylor W.G., Allen M.E., Kitas G.D., Bacon P.A., Young S.P. (1996). Characterization of altered calcium signalling in T lymphocytes from patients with rheumatoid arthritis (RA). Clinical and Experimental Immunology.

[28] Partida-Sánchez S., Cockayne D.A., Monard S., Jacobson E.L., Oppenheimer N., Garvy B. (2001). Cyclic ADP-ribose production by CD38 regulates intracellular calcium release, extracellular calcium influx and chemotaxis in neutrophils and is required for bacterial clearance in vivo. Nature Medicine.

[29] Malavasi F., Deaglio S., Funaro A., Ferrero E., Horenstein A.L., Ortolan E. (2008). Evolution and function of the ADP ribosyl cyclase/CD38 gene family in physiology and pathology. Physiological Reviews.

[30] Hartiala P., Hytönen J., Pelkonen J., Kimppa K., West A., Penttinen M.A. (2007). Transcriptional response of human dendritic cells to Borrelia garinii--defective CD38 and CCR7 expression detected. Journal of Leukocyte Biology.

[31] Suliman H.B. (2004). Lipopolysaccharide induces oxidative cardiac mitochondrial damage and biogenesis. Cardiovascular Research.

[32] Garrabou G. (2012). The effects of sepsis on mitochondria. Journal of Infectious Diseases.

[33] Hahn W.S., Kuzmicic J., Burrill J.S., Donoghue M.A., Foncea R., Jensen M.D. (2014). Proinflammatory cytokines differentially regulate adipocyte mitochondrial metabolism, oxidative stress, and dynamics. The American Journal of Physiology – Endocrinology and Metabolism.

